# Evidence of Polymorphism on the Antitrypanosomal Naphthoquinone (4*E*)-2-(1*H*-Pyrazol-3-ylamino)-4-(1*H*-pyrazol-3-ylimino)naphthalen-1(4*H*)-one

**DOI:** 10.3797/scipharm.1209-10

**Published:** 2013-06-04

**Authors:** Norma R. Sperandeo, Sonia N. Faudone

**Affiliations:** 1 Departamento de Farmacia, Facultad de Ciencias Químicas, Universidad Nacional de Córdoba, Haya de la Torre y Medina Allende, Ciudad Universitaria, X5000HUA, Córdoba, Argentina; 2 Centro de Excelencia en Productos y Procesos de Córdoba (CEPROCOR), Álvarez de Arenales 230, X5004AAP Córdoba, Argentina.

**Keywords:** Pyrazolylnaphthoquinones, Chagas disease, Polymorphism, Powder X-ray Diffraction, DSC, TG

## Abstract

The aim of this study was to characterize the solid state properties of (4*E*)-2-(1*H*-pyrazol-3-ylamino)-4-(1*H*-pyrazol-3-ylimino)naphthalen-1(4*H*)-one (**BiPNQ**), a compound with a significant inhibitory activity against *Trypanosoma cruzi,* the etiological agent of Chagas disease (American trypanosomiasis). Methods used included Differential Scanning Calorimetry (DSC), Thermogravimetry (TG), Fourier Transform Infrared Spectroscopy (FTIR), Powder X-Ray Diffraction (PXRD), Hot Stage, and Confocal Microscopy. Two **BiPNQ** samples were obtained by crystallization from absolute methanol and 2-propanol-water that exhibited different thermal behaviours, PXRD patterns, and FTIR spectra, indicating the existence of an anhydrous form (**BiPNQ-I**) and a solvate (**BIPNQ-s**), which on heating desolvated leading to the anhydrous modification **BiPNQ-I**. It was determined that FTIR, DSC, and PXRD are useful techniques for the characterization and identification of the crystalline modifications of **BiPNQ**.

## Introduction

**Fig. 1:**
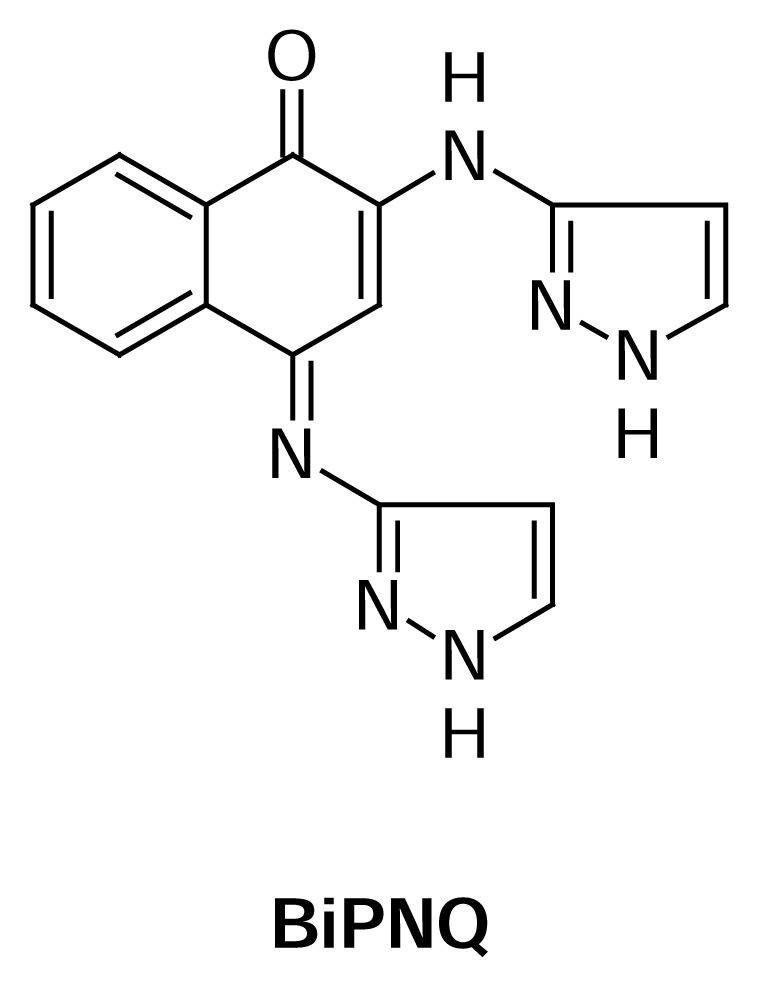
Chemical structure of (4*E*)-2-(1*H*-pyrazol-3-ylamino)-4-(1*H*-pyrazol-3-ylimino)-naphthalen-1(4*H*)-one (**BiPNQ**).

Many organic and inorganic compounds are able to exist in more than one crystalline form, a property known as polymorphism [1–8]. The relevance of this phenomenon in the pharmaceutical industry is that the different polymorphs of an active pharmaceutical ingredient (API) generally exhibit different melting points, X-ray powder patterns, solubility, and bioavailability, even though they are chemically equivalent [1–8]. Different polymorphs also lead to distinct morphology, tensile strength, and density of powders which all contribute to the compression characteristics of materials, which can have a direct effect on the ability to process and/or manufacture the drug substance and the drug product [5–7]. Thus, knowledge of the physical forms of an API is critical for the performance of a solid dosage form and the success or failure of a pharmaceutical product [3, 5, 7, 8].

(4*E*)-2-(1*H*-Pyrazol-3-ylamino)-4-(1*H*-pyrazol-3-ylimino)naphthalen-1(4*H*)-one (**BiPNQ**, Figure 1) is a compound that exhibits good inhibitory activity against *Trypanosoma cruzi*[9], the causative agent of American trypanosomiasis (Chagas disease), a disease with 15–20 million chronically infected carriers in the tropics and subtropics of North and South America, and more than 100 million people at risk [10]. Current estimates indicate 200,000 new cases every year and an annual mortality of 50,000 [11]. The Chagas disease is one of the many parasitic illnesses existing today, for which no satisfactory pharmacological treatment exists. The nitroheterocyclic drugs nifurtimox and benznidazole were introduced in 1965 and 1971, respectively, and are at present the only drugs that proved to have efficacy to combat *T. cruzi* in the acute stage of the illness; however, both compounds provoke serious lateral effects in the host and their use has led to parasitic resistance. Nifurtimox was retired from the Argentinian, Chilean, Brazilian, Paraguayan, and Bolivian markets various years ago [12]; thus, only benznidazole is used in these countries, but it is not easily available. Taking into account that **BiPNQ** was shown to have an anti-*T. cruzi* activity slightly better than that of benznidazole [9], it is important to examine the aspects of its solid state properties to support the development effort.

In the present study, two solid samples of **BiPNQ** were obtained by crystallization experiments and were characterized by means of Differential Scanning Calorimetry (DSC), Termogravimetry (TG), Hot Stage and Confocal Microscopy, Fourier Transform Infrared Spectroscopy (FTIR), and Powder X-Ray Diffraction (PXRD) in order to evidence their differences. In addition, the product of desolvation of one of the prepared **BiPNQ** samples was identified by PXRD, and its DSC, TG, and FTIR profiles are presented.

## Results and Discussion

### DSC, TG, Hot Stage, and Confocal Microscopy

**Fig. 2:**
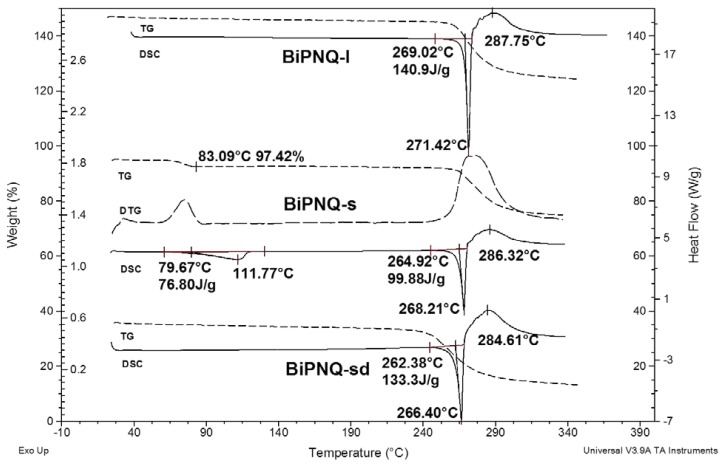
Overlaid DSC (crimped pan) and TG (open pan) curves (10 °C/min and flowing N_2_ at 50 mL/min) of **BiPNQ** samples. **BiPNQ-I** (recrystallized from absolute methanol, upper); **BiPNQ-s** (recrystallized from 2-propanol-water, middle) and the sample obtained after drying a portion of **BiPNQ-s** in vacuum at 60 °C (**BiPNQ-sd**, lower). The DTG curve of **BiPNQ-s** was included for a better vissualization of the TG desolvation step.

Figure 2 shows the DSC and TG curves of **BiPNQ-I**, **BiPNQ-s** and **BiPNQ-sd** (the fully desolvated **BiPNQ-s** sample). As can be seen, **BiPNQ-I** and **BiPNQ-s,** the two crystallized samples, showed differences in their thermal behaviors.

For example, **BiPNQ-I** was a solvent-free solid, as neither DSC desolvation peaks nor TG weight losses were observed in the 25–200 °C range (Figure 2 upper). The DSC scan displays a single sharp endothermic peak at 269.0 °C (extrapolated onset temperature, T_e_), superimposed with a large exothermic effect centered at 287.8 °C. According to the respective TG curve, both effects had associated weight loss, indicating melting with the decomposition process. The thermal events just described were supported by observations made using a Kofler apparatus. By heating from 25 up to 250 °C, phase modifications or evaporation losses were not produced, which is consistent with the DSC and TG results. At about 270 °C, the red crystalline particles of **BiPNQ-I** melted and upon further heating the molten phase diminished in size and darkened, typical of a decomposition process [13, 14]. Consequently, after cooling the slide, a black solid residue was obtained.

In the case of **BiPNQ-s,** the DSC trace (Figure 2 middle) showed two endothermic peaks. The first one, which was broad and had a T_e_ of 79.7 °C, was attributed to the loss of solvent from the sample since the respective TG-DTG curves showed a step (TG) and a peak (DTG) in this temperature range. The experimental weight loss of 2.6% is in agreement with the calculation for a hemihydrate of 2.8%. The second peak was sharp and was superimposed with a broad exothermic effect, representing melting with the decomposition process that started at 264.9 °C (T_e_). Taking into account that desolvation peaks are affected by the DSC experimental conditions [15, 16], the DSC curve of **BiPNQ-s** was also obtained in static air (curve not shown). As expected, the desolvation temperature measured in static air was a little higher than that in flowing N_2_ (Figure 2), due to a slow removal of the solvent vapor generated [15], but the melting temperature was not affected.

The effect of desolvation on the thermal behaviour of **BiPNQ-s** was also investigated. Accordingly, a portion was heated in a desiccant pistol with CaCl_2_ at 60 °C during 4 hours in vacuum. After that, the sample was allowed to cool at 25 °C inside the pistol, and its DSC and TG curves were obtained. As shown in Figure 2 (lower), neither DSC desolvation peaks nor TG weight losses were observed in the 25–200 °C range, indicating that a free-solvent solid was obtained. The DSC profile was similar to that of **BiPNQ-I** (Figure 2 upper), but the melting peak (T_e_ = 262.4 °C) and the decomposition one were downshifted, suggesting that a new anhydrous phase was obtained. However, as it is described below, this fully desolvated sample (**BiPNQ-sd**) was not a new anhydrous form of **BiPNQ**. On the contrary, it was isostructural to **BiPNQ-I**. Thus, the downshifting of the DSC peaks can be attributed to a low degree of crystallinity of the resulting solid and/or to external factors (morphology, size, and crystal defects) of the lattice structure [17].

**Fig. 3:**
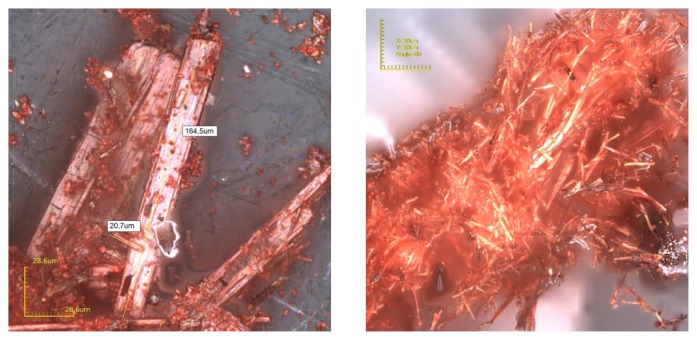
Confocal images (laser confocal microscope) of **BiPNQ-I** (left, magnification: 1428x) and **BiPNQ-sd** (right, magnification: 2132x). The scales were 28.6 × 28.6 μm (**BiPNQ-I**) and 20 × 20 μm (**BiPNQ-sd**).

In order to obtain information about the morphology and crystallite size of **BiPNQ-I** and **BiPNQ-sd**, microscopic observations (laser confocal microscope) were performed (Figure 3). As shown in Figure 3 left, **BiPNQ-I** was a mixture of red prisms of approximately 160 μm long and red particles irregular in shape and size, while **BiPNQ-sd** (Figure 3 right) consisted of aggregated needle-like crystals of approximately 15–35 μm long. Thus, the crystal size of **BiPNQ-sd** was smaller than that of **BiPNQ-I,** and this evidently contributed to the downshifting of the melting and decomposition temperatures of **BiPNQ-sd** as DSC temperature data depend on particle size factors [18].

### Powder X-Ray Diffraction (PXRD) and Fourier Transform Infrared Spectroscopy (FTIR)

**Fig. 4:**
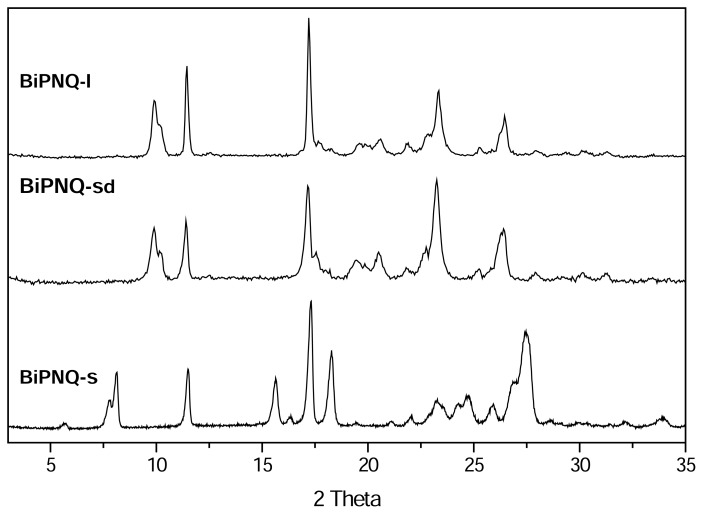
Powder XRD patterns of **BiPNQ-I** (upper), **BiPNQ-s** (lower) and the sample obtained after drying **BiPNQ-s** in vacuum at 60 ºC (**BiPNQ-sd,** middle).

The PXRD patterns of **BiPNQ-I** and **BiPNQ-s** are depicted in Figure 4. As can be seen, the patterns showed differences in the position of various reflections, indicating that **BiPNQ-I** and **BiPNQ-s** were not isomorphous.

For example, **BiPNQ-I** (Figure 4 upper) showed the most intense peak at 17.2° 2Θ and characteristic lines at 9.9°, 10.3°, 11.4°, 23.3°, and 26.5° 2Θ. In contrast, **BiPNQ-s** (Figure 4 lower) exhibited the most intense peak at 17.3° and characteristic peaks at values of 7.8°, 8.1°, 11.5°, 15.6°, 18.3°, 26.9°, and 27.5° 2Θ. Thus, these observations indicated that **BiPNQ-I** and **BiPNQ-s** represented different crystalline structures.

**Fig. 5:**
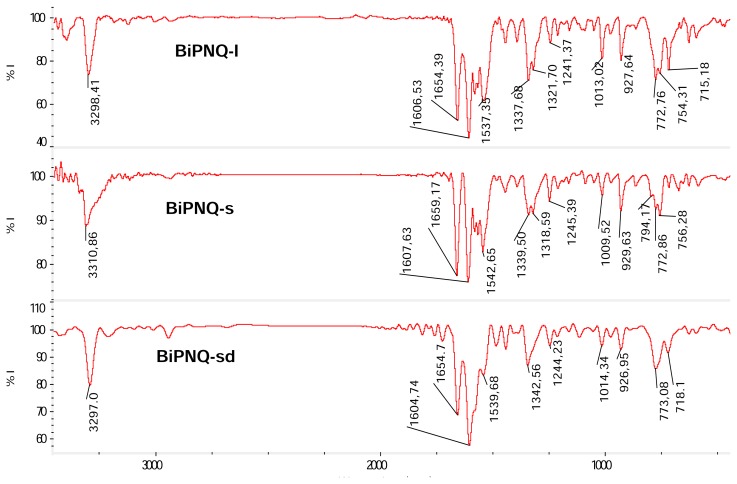
FTIR spectra (KBr pellets) of **BiPNQ-I** (upper), **BiPNQ-s** (middle) and the sample obtained after drying **BiPNQ-s** in vacuum at 60 °C (**BiPNQ-sd**, lower).

The IR spectra of **BiPNQ-I** and **BiPNQ-s** (Figure 5, upper and middle) also showed differences in the positions and relative intensities of various bands, particularly in the N-H stretching region, the double bond stretch vibrations, and the aromatic bending regions, indicating differences in their crystal lattices, and this is consistent with the PXRD results. For instance, **BiPNQ-I** showed a medium sharp band at 3298 cm^−1^ due to the three NH groups of the molecule, whereas in **BiPNQ-s** this band is broad and was centered at 3311 cm^−1^.

Regarding the desolvation of **BiPNQ-s**, PXRD analysis revealed that by the effect of heating (60 °C, vacuum under CaCl_2_) a phase change occurred, which led to the formation of **BIPNQ-I**. In fact, the PXRD pattern (Figure 4 middle) of the fully desolvated sample **(BiPNQ-sd**) was different from that of **BiPNQ-s** (Figure 4 lower), but it matched that of **BiPNQ-I** (Figure 4 upper). In comparing the diffractogram of **BiPNQ-s**d with the one of **BiPNQ-I,** it was also evident that the former exhibited some peak broadening. In particular, the peaks at 17.2°, 23.3°, and 26.4° 2Θ were much broader in **BiPNQ-sd** than in **BiPNQ-I,** which is an indication of the different morphology/crystal size/degree of crystallinity in the two samples and this is in accordance with the microscopic data that revealed differences in particle size and crystalline habits for **BiPNQ-s**d and **BiPNQ-I.** The IR spectrum of **BiPNQ-sd** (Figure 5 lower) was very similar to that of **BIPNQ-I** and this confirmed the PXRD results. Thus, **BiPNQ-s** is a solvate that did not retain its crystal structure after desolvation, and it may be considered a stoichiometric solvate according to the Griesser classification [19], since this class of solvates always lead to a different crystal structure or results in an amorphous or disordered phase.

In conclusion, the physical characterization of the two **BiPNQ** samples with thermal, microscopic, spectroscopic, and powder X-ray diffraction methods was accomplished. The results indicated that **BiPNQ** exists at least in one anhydrous form and one solvate, which have characteristic IR spectra and PXRD patterns useful to fingerprint them. The fact that very similar solvents such as absolute methanol and isopropanol yielded different polymorphs strongly suggests that other more dissimilar solvents may also yield additional modifications; therefore, a more extensive search for polymorphs of **BiPNQ** is warranted.

## Experimental

### Materials

(4*E*)-2-(1*H*-Pyrazol-3-ylamino)-4-(1*H*-pyrazol-3-ylimino)naphthalen-1(4*H*)-one (**BiPNQ**, MW 304.11) was prepared and purified to analytical grade according to a previously reported method [9]. On the basis of solubility characteristics and crystallization yield considerations, solid samples were prepared according to the following procedures: a) the dry powder was dissolved in boiling absolute methanol to obtain a saturated solution of the drug. After filtration, the solution was allowed to cool first to 25 °C (room temperature, RT) and then to −20 °C until crystals were formed; and b) the dry powder was dissolved in boiling 2-propanol to obtain a saturated solution. After that, water was added drop by drop until a precipitate was obtained. All the obtained crystals were isolated by vacuum filtration and washed with cold absolute methanol or 2-propanol. In the following sections, the solid samples will be referred to as: **BiPNQ-I** (absolute methanol) and **BiPNQ-s** (2-propanol-water). The purity of the crystals was checked by Thin Layer Chromatography (TLC), finding that all the samples were chromatographically pure. For TLC analysis, pre-coated silica gel 60 F_254_ (Merck) plates were used and spots were visualized by UV and daylight. Solvents of analytical reagent grade were employed.

### DSC, TG, Hot Stage, and Confocal Microscopy

DSC and TG measurements were recorded on MDSC 2920 and TG 2950 analysers (TA Instruments Inc., New Castle, USA), respectively, in the temperature range 20–25 °C and 375 °C under nitrogen purge (99.99% purity, flow rate of 50 mL/min) and at a heating rate of 5 or 10 °C/min. The DSC (crimped pan system) and TG temperature axes were calibrated by using indium (99.99% purity, melting point. 156.6 °C) and Ni (99.99% purity, Curie point 358.3 °C) as standards, respectively. Empty aluminum pans were used as references. Samples of approximately 2 mg were weighed using a Cahn-33 microbalance. The DSC and TG curves were processed with Universal Analysis 2000 software (TA Instrument, Inc.).

The physical and morphological changes of the samples that occurred during heating were observed through a microscope fitted with a Kofler hot-stage (Leitz, Wetzlar, Germany) at a constant rate from RT (at about 8 °C/min) up to 280 °C. In order to provide experimental conditions similar to that of the DSC measurements, the samples were not embedded in silicone oil.

The particle size and morphological features of the samples were examined with a laser confocal microscope (Olympus LEXT OLS4000 3D Laser Measuring Microscope, Olympus Corp., Japan).

### PXRD and FTIR

The PXRD pattern of **BiPNQ-I** and those of **BiPNQ-s** and **BiPNQ-sd** (fully desolvated) samples were collected at RT on the D8 ADVANCE (Bruker AXS, Germany) X-ray powder diffractometer fitted with a Copper tube (Cu Kα radiation λ = 1.5418 Å) and a post-diffraction graphite monochromator. The X-ray generator was set at a voltage of 40 kV and a current of 40 mA. Samples were subject to PXRD analysis in step mode with a step size of 0.05° 2Θ and a step time of 30 s over an angular range of 2–50° 2Θ. The sample holder was rotated in a plane parallel to its surface at the speed of 30 rpm during the measurements.

The FTIR spectra (4000–400 cm^−1^) were recorded on a Nicolet 5-SXC spectrophotometer (Nicolet Instruments Corporation, Madison, WI). KBr pellets of solid samples were prepared with a mini-press (Hidráulicos Delfabro, Argentina) at 6 tons without any extra grinding. About 1 mg of sample was dispersed in 200 mg of dry KBr. The spectra were collected with 40 scans at 8 cm^−1^ resolution, and processed with the OMNIC 1.1 program (Nicolet). KBr scans were used as the background.
